# Deciphering the contributions of cuproptosis in the development of hypertrophic scar using single-cell analysis and machine learning techniques

**DOI:** 10.3389/fimmu.2023.1207522

**Published:** 2023-06-20

**Authors:** Binyu Song, Wei Liu, Yuhan Zhu, Yixuan Peng, Zhiwei Cui, Botao Gao, Lin Chen, Zhou Yu, Baoqiang Song

**Affiliations:** Department of Plastic Surgery, Xijing Hospital, Fourth Military Medical University, Xi’an, Shaanxi, China

**Keywords:** hypertrophic scar, cuproptosis, single-cell, machine learning, GEO

## Abstract

Hypertrophic scar (HS) is a chronic inflammatory skin disease characterized by excessive deposition of extracellular matrix, but the exact mechanisms related to its formation remain unclear, making it difficult to treat. This study aimed to investigate the potential role of cuproptosis in the information of HS. To this end, we used single-cell sequencing and bulk transcriptome data, and screened for cuproptosis-related genes (CRGs) using differential gene analysis and machine learning algorithms (random forest and support vector machine). Through this process, we identified a group of genes, including ATP7A, ULK1, and MTF1, as novel therapeutic targets for HS. Furthermore, quantitative real-time polymerase chain reaction (qRT-PCR) was conducted to confirm the mRNA expression of ATP7A, ULK1, and MTF1 in both HS and normal skin (NS) tissues. We also constructed a diagnostic model for HS and analyzed the immune infiltration characteristics. Additionally, we used the expression profiles of CRGs to perform subgroup analysis of HS. We focused mainly on fibroblasts in the transcriptional profile at single-cell resolution. By calculating the cuproptosis activity of each fibroblast, we found that cuproptosis activity of normal skin fibroblasts increased, providing further insights into the pathogenesis of HS. We also analyzed the cell communication network and transcription factor regulatory network activity, and found the existence of a fibroblast-centered communication regulation network in HS, where cuproptosis activity in fibroblasts affects intercellular communication. Using transcription factor regulatory activity network analysis, we obtained highly active transcription factors, and correlation analysis with CRGs suggested that CRGs may serve as potential target genes for transcription factors. Overall, our study provides new insights into the pathophysiological mechanisms of HS, which may inspire new ideas for the diagnosis and treatment.

## Introduction

Hypertrophic scar (HS) is a result from chronic inflammation in the reticular dermis and is an abnormal scar that forms as a result of improper wound healing (1). This condition occurs when there is an excessive production of collagen proteins in response to skin injuries such as burns or surgical incisions (2). HS is a skin lesion that results from excessive proliferation of newly formed connective tissue in the dermis or deeper tissues, where fibroblasts play a key role (3, 4). HS is usually limited to the boundaries of the original wound and may cause physical and psychological discomfort, including itching, pain, and functional loss (5). Overall, the formation of HS is a complex process involving multiple cell types and molecular mechanisms. Although various treatment options for HS exist, such as surgery, steroid injections, laser and skin grafting, there is currently no guaranteed cure. The current focus of research is to understand the molecular mechanisms of HS formation and to identify new therapeutic approaches for preventing or treating HS.

Excessive proliferation and activation of fibroblasts are characteristics of fibrosis, and promoting cell apoptosis appears to be an effective solution for preventing tissue scarring and reversing fibrosis that has already formed (6, 7). Ferroptosis, as another form of programmed cell death, has been shown to be involved in alleviating fibrotic diseases (8, 9). Cuproptosis is a newly discovered form of programmed cell death that is directly linked to the acylation of components of the tricarboxylic acid (TCA) cycle by copper, resulting in toxic protein stress and ultimately cell death (10). Cuproptosis has been shown to be potentially involved in alleviating pulmonary fibrosis, but its role in dermal fibroblasts has not yet been studied (11). Currently, little is known about the role of cuproptosis in HS.

The emergence of single-cell RNA sequencing (scRNA-seq) technology provides unprecedented molecular information and is the most important methodological advance and breakthrough, playing a critical role in exploring various disease mechanisms (12). In this study, through the integration of single-cell sequencing with bulk transcriptome sequencing, we investigated for the first time the relationship between the occurrence and development of HS and cuproptosis, deepening our understanding of the underlying mechanisms of HS and providing new emerging potential therapeutic strategies and research foundations for inhibiting scar formation.

## Materials and methods

### Data acquisition

The Gene Expression Omnibus (GEO) is a database created by the NCBI that stores a large amount of gene expression data. We obtained the dataset GSE181540 from GEO (https://www.ncbi.nlm.nih.gov/geo/), which includes 3 HS and 3 NS tissues, as well as 6 NS tissues from GSE158395, 3 NS tissues from GSE190626, and 3 HS tissues from GSE210434. In addition, we obtained dataset OEP002674 from The National Omics Data Encyclopedia (NODE, https://www.biosino.org/), which includes 5 HS and 5 NS samples. Based on previous studies on cuproptosis-related genes (CRGs), we identified a set of 56 genes related to cuproptosis (10, 13–15).

### Data processing and differential gene expression analysis

We annotated, normalized, and performed log2 transformation on different datasets using R. Then, we used the “sva” R package to merge the aforementioned datasets, correct batch effects, and extract CRGs from the merged dataset. We performed differential gene analysis on the processed dataset using the “limma” package and screened for differentially expressed genes. The threshold was set to |log2 Fold change (FC)| > 1 and p-value <0.05.

### Functional and pathway enrichment analysis

We performed Gene Ontology (GO) enrichment analysis on the genes from three aspects: biological process, cellular component, and molecular function, in order to identify the biological functions of the genes. Kyoto Encyclopedia of Genes and Genomes (KEGG) is a primary public database related to pathways. We used the hypergeometric test to identify significantly enriched pathways in the genes. We performed functional and pathway enrichment analysis on the differentially expressed genes using these two methods, and set the P-value threshold to 0.05.

### Machine learning

We used the “randomForest” R package to perform Random Forest (RF) feature selection on the dataset. The RF algorithm is an ensemble learning method that uses multiple decision trees to form a regressor. It is used to screen for the importance of genes in diseases. Its main advantages are its good performance in processing high-dimensional data and insensitivity to outliers. Then, we used the “e1071” R package to perform support vector machine-recursive feature elimination (SVM-RFE) feature selection on the gene set. SVM-RFE scores and ranks gene features using binary classification and selects the top few genes with the lowest error rate. We took the intersection of the results from these two machine learning methods and the differentially expressed genes that we previously screened for, and used the 11 intersected genes as hub genes for further analysis.

### Differential validation of hub genes in external dataset

We selected 5 HS samples and 3 NS samples from the dataset GSE188952 in GEO to perform external differential expression validation of the hub genes. We calculate the differences and significance of the genes between the disease group and the control group, and plotted the gene differential boxplot. We selected the genes that were validated and further analyzed them.

### Construction and evaluation of diagnostic model

The three selected genes were subjected to multivariable logistic regression modeling. Using the generalized linear model with binary classification, the entire dataset was used as the training set to construct the model. ROC curve was plotted and the area under the curve (AUC) was calculated. Finally, bootstrap validation was performed with 1000 resampling iterations to calculate the sensitivity and specificity of the model. Further evaluation of the stability and reliability of the model was conducted through construction of nomogram and decision curves.

### Immune infiltration and inflammation factor analysis

Previous studies have demonstrated a significant association between cuproptosis and cellular immunity (16–18). In this study, we performed a Pearson correlation analysis using the corrplot R package to investigate the relationship between the mRNA expression of the 3 CRGs. Additionally, we used the “IOBR” R package to apply the CIBERSORT and MCPcounter algorithms for quantifying the degree of immune cell infiltration in the samples (19). We also analyzed the correlation between the 3 CRGs and immune cell infiltration as well as the expression of inflammatory factors.

### Consensus clustering analysis

We utilized the “ConsensusClusterPlus” R package to perform a k-means consensus clustering analysis, aiming to identify distinct subtypes that are associated with the expression of CRGs.

### Functional enrichment analysis between two subtypes

The differentially expressed genes(|log2 FC|>1, p-value<0.05) identified between two subtypes was used for GO biological function enrichment analysis and KEGG pathway enrichment analysis by using R package “clusterprofiler” and “enrichplot”

### Single-cell RNA statistical processing

Single-cell sequencing dataset analysis has been used to explore a variety of skin diseases with promising results (20–22). In our study, we used the “Seurat” R package to create a Seurat object and normalize and scale cells. By excluding cells with less than 200 expressed genes, more than 2500 expressed genes, or mitochondrial gene content higher than 10%, we retained 45095 cells. We selected the top 3000 highly variable genes for principal component analysis with a setting of 15 principal components for subsequent dimensionality reduction and clustering. We integrated samples and removed batch effects using the “harmony” function, obtained unsupervised cell clusters using a graph-based clustering method (with a resolution of 0.5), and visualized them using t-SNE plots. We used the “FindAllMarkers” function and Wilcoxon rank-sum test algorithm to identify marker genes for each cell cluster, with conditions including |log2 FC|>0.25, P<0.05, and a minimum percentage>0.1. To better identify fibroblast clusters, we selected clusters of fibroblast cell types for further t-SNE analysis, graph-based clustering, and marker gene analysis. We calculated the score of the cuproptosis in each cell using the “AddModuleScore” function and divided cells into high and low groups based on the median score.

### Cell-cell communication analysis

We used the recently developed tool “CellChat” R package to perform comprehensive analysis of cell-cell communication molecules. This tool can generate and plot the probability and interaction strength of cell-cell communication from single-cell transcriptome data, allowing for in-depth analysis of cell-cell communication. The normalized count and cell types by Seurat were used for this analysis.

### Scenic transcription factor regulatory network analysis

The SCENIC transcription factor inference was performed using the R SCENIC package following the recommended pipeline steps (23).

### Patient and ethical declaration

We collected 3 NS tissues and 3 HS tissues. Prior to surgery, we provided patients with a detailed explanation of the research purpose and process, and obtained their consent to participate in the study. We also obtained written informed consent from all patients or their legal guardians and received approval from the Ethics Committee of the Xijing Hospital, Fourth Military Medical University.

### Real-time quantitative polymerase chain reaction

The tissues were subjected to RNA extraction using the TRIzol reagent (Invitrogen, Camarillo, CA, United States). The extracted RNA was then reverse transcribed into cDNA using the PrimeScript RT reagent Kit with gDNA Remover (Takara, Shiga, Japan) at a concentration of 1,000 ng. For qRT-PCR, the TB Green Premix Ex Taq II (Takara, Shiga, Japan) was utilized, and the BIO-RAD CFX Connect Real-Time System (Bio-Rad, Munich, Germany) was used for the analysis. The expression levels of target genes were normalized to GAPDH. Forward 5′-GCACCGTCAAGCTGAGAAC-3′ and reverse 5′-TGGTGAAGACGCCAGTGGA-3′ for human GAPDH, forward 5′-GGCAAGTTCGAGTTCTCCCG-3′ and reverse 5′-CGACCTCCAAATCGTGCTTCT-3′ for human ULK1, forward 5′-CAGTGCGGAGAACACTTGC-3′ and reverse 5′-TGCACATAACCCTGGGACATT-3′ for human MTF1, forward 5′-TGGGACCATATCCAAAGCACA-3′ and reverse 5′-CAAGCTCCTGACTTTTTCTCCAG-3′ for human ATP7A.

## Results

### Differential gene expression analysis

**Figure 1:**
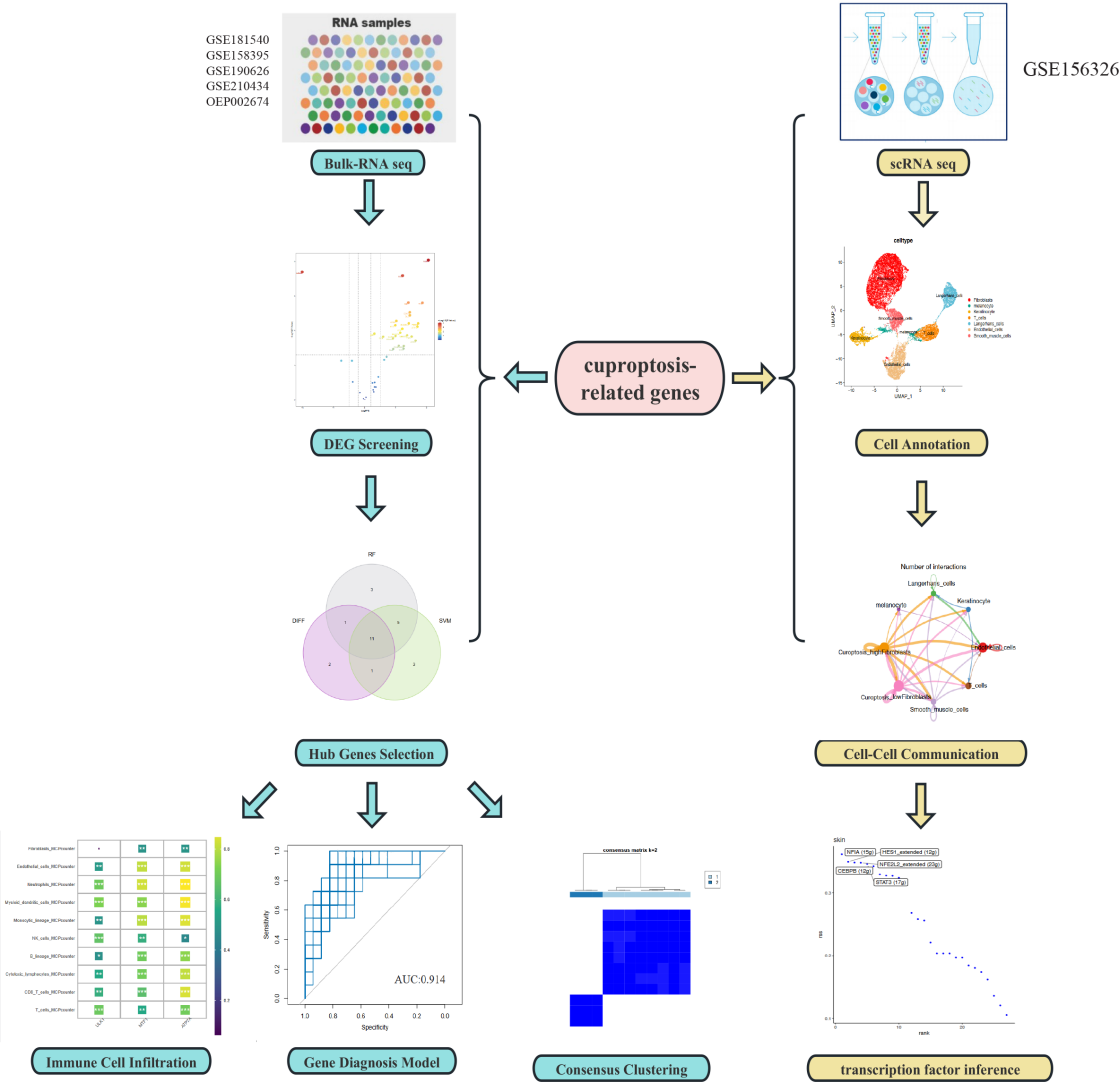
Flowchart of the study.

**Figure 2:**
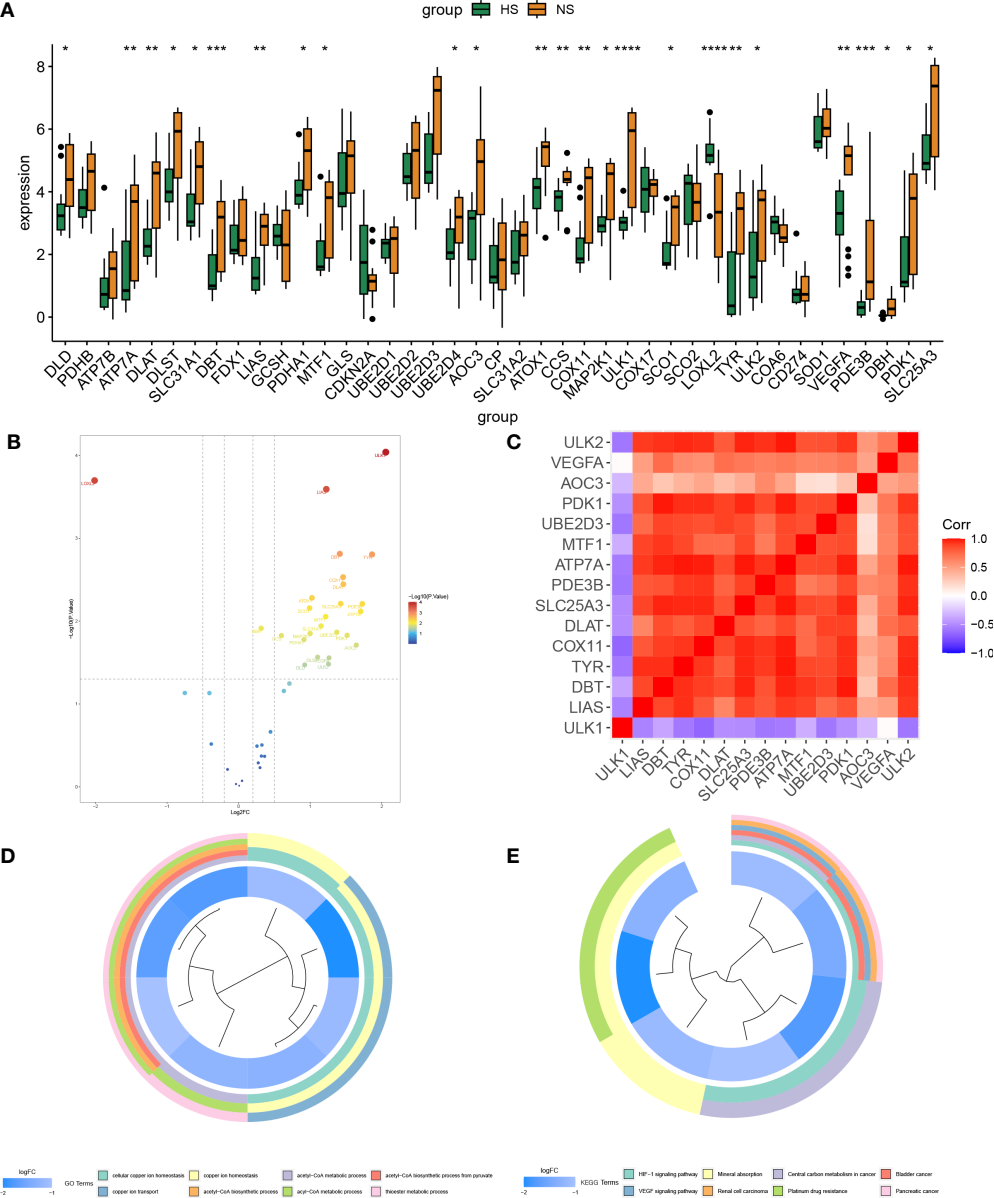
Screening and correlation analysis of differentially expressed genes and their functional enrichment analysis. **(A, B)** Differential box plot and volcano plot of CRGs in HS. **(C)** Heat map of correlation between differentially expressed genes. **(D, E)** GO, KEGG enrichment analysis of differentially expressed genes, different colors represent various significant pathways and related enriched genes. (*P < 0.05, **P < 0.01, ***P < 0.001, ****P < 0.0001).

The research process of this study can be seen from the graphic flow chart (**Figure 1**). The “Limma” R package was used to identify differentially expressed CRGs in the dataset. The filtering criteria were set as |log2 FC| > 1 and p-value <0.05, and boxplots and volcano plots of the differentially expressed genes were generated (**Figures 2A, B**). **Figure 2C** shows the correlation between differentially expressed genes. Among the 56 CRGs, 15 genes were found to be significantly differentially expressed, including 14 downregulated genes and 1 upregulated gene.

### Enrichment analysis of differentially expressed genes

The differentially expressed genes were subjected to enrichment analysis using the GO and KEGG methods, and enrichment circle plots were generated for both (**Figures 2D, E**). In GO analysis, the genes were mainly enriched in cellular copper ion homeostasis, copper ion transport, and copper ion homeostasis, while in KEGG analysis, the genes were mainly enriched in the HIF-1 signaling pathway, VEGF signaling pathway, and Mineral absorption pathways.

### Machine learning-based selection of hub genes

**Figure 3:**
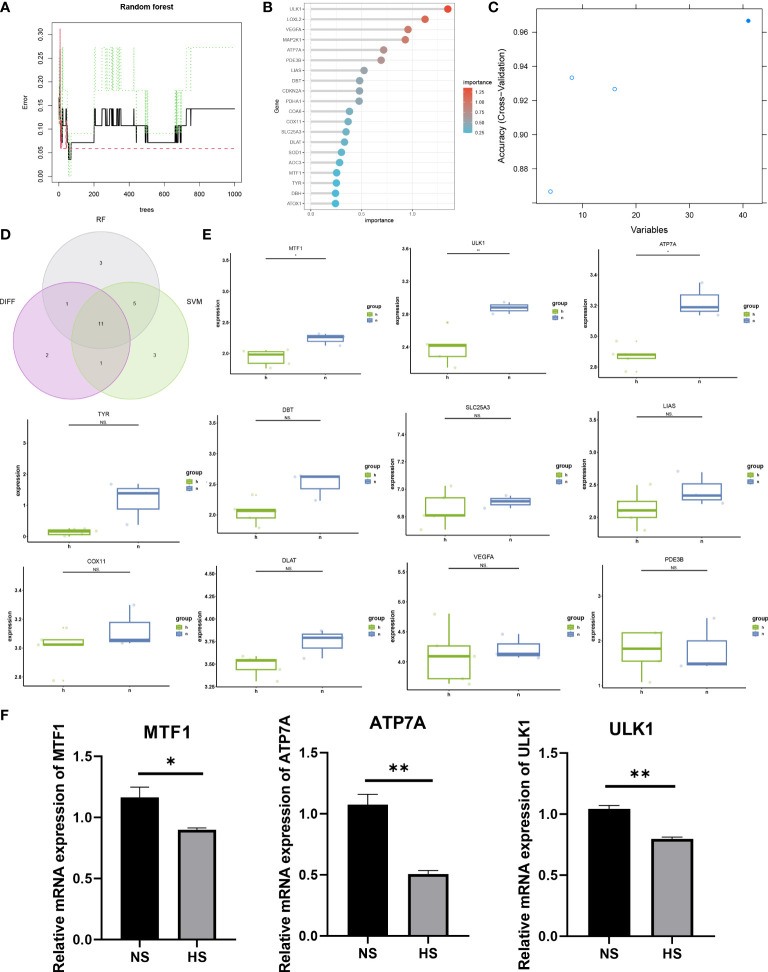
Screening of candidate hub genes and validation of differential expression. **(A, B)** Ranking the importance of genes using the random forest algorithm. **(C)** SVM-REF algorithm selects genes with the highest accuracy. **(D)** The intersection genes of the top 20 genes of importance in the RF algorithm, the top 20 genes with the highest accuracy in the SVM-REF algorithm, and differentially expressed genes. **(E)** Validation of differential expression of candidate hub genes in external dataset. **(F)** Differential expression validation using qRT-PCR for candidate hub genes. (*p < 0.05, **p < 0.01 and ns = not significant).

Machine learning methods are widely used in dermatological diseases (24–26). The SVM-RFE and RF machine learning methods were used to further screen candidate hub genes. First, the RF algorithm was used to rank the importance of each gene (**Figures 3A, B**), and the top 20 genes with the highest importance ranking were selected: ULK1, LOXL2, VEGFA, MAP2K1, ATP7A, PDE3B, LIAS, DBT, CDKN2A, PDHA1, COA6, COX11, SLC25A3, DLAT, SOD1, AOC3, MTF1, TYR, DBH, ATOX1. Then, the top 20 genes with the highest accuracy in the SVM-RFE results were selected: LOXL2, ULK1, DBT, PDE3B, LIAS, COX11, TYR, CCS, ATP7A, ATOX1, VEGFA, DLAT, SCO1, PDK1, MAP2K1, SLC25A3, PDHA1, DLST, DBH, MTF1 (**Figure 3C**). The intersection of the two machine learning methods’ results was taken as the candidate hub genes for further analysis.

### Differential gene validation using external dataset

The candidate hub genes selected using machine learning and the 15 significantly differentially expressed genes obtained from differential analysis were intersected to obtain 11 genes (**Figure 3D**): MTF1, ULK1, ATP7A, TYR, DBT, SLC25A3, LIAS, COX11, DLAT, VEGFA, PDE3B. The external dataset GSE188952 was used for validation of differential gene expression (**Figure 3E**), and 3 CRGs with significant differential expression in the external dataset were identified: MTF1, ULK1, ATP7A.

### Validation of the expression of three cuproptosis-related mRNA in HS and NS

To further verify the expression levels of the three CRGs, we collected specimens from 3 patients with NS and 3 HS with patients. The mRNA expression level of MTF1, ULK1 and ATP7A in NS was significantly higher than that in HS, which also consistent with the expression of bulk RNA-seq datasets (**Figure 3F**).

### Establishment and evaluation of the diagnostic model

**Figure 4:**
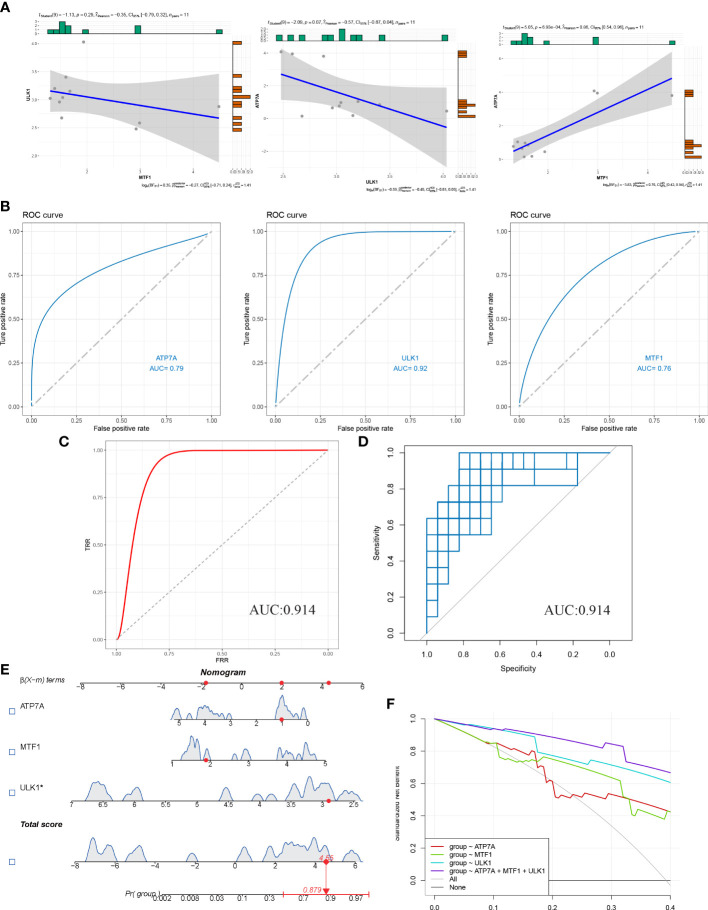
Diagnostic model of HS was constructed and evaluated. **(A)** Correlation analysis of three candidate hub genes. **(B)** Nomogram of candidate hub genes in the hyperplastic scar dataset and the area under the curve (AUC). **(C)** ROC curve and AUC using SVM-REF and DEGs diagnostic models. **(D)** Bootstrap resampling algorithm to verify the model. **(E)** Prediction of hypertrophic scarring using nomogram. **(F)** The decision curve evaluates the predictive performance of the model.

Firstly, a correlation graph between 3 CRGs was drawn (**Figure 4A**), and ROC curve was plotted to evaluate the diagnostic specificity and sensitivity of each gene, and the area under the curve (AUC) was calculated. ATPA (AUC=0.79), ULK1 (AUC=0.92), and MTF1 (AUC=0.76) were obtained (**Figure 4B**). We performed multivariable logistic regression modeling on three hub genes, and the model was evaluated using ROC curve (**Figure 4C**). The AUC was calculated to be 0.914. Further verification was performed using the bootstrap algorithm with R=1000 resampling times, and the average AUC value was0.879893, with a 95% confidence interval (CI) of 0.7303-0.9189 (**Figure 4D**). These results indicate that the diagnostic model has high diagnostic value. A nomogram of the model was drawn, and the total score of the sample was calculated (**Figure 4E**). Finally, the Decision Curve Analysis (DCA) curve was plotted (**Figure 4F**), and it showed that the diagnostic model composed of MTF1, ULK1, and ATP7A had the highest diagnostic performance.

### Immune infiltration and inflammatory factor analysis

**Figure 5:**
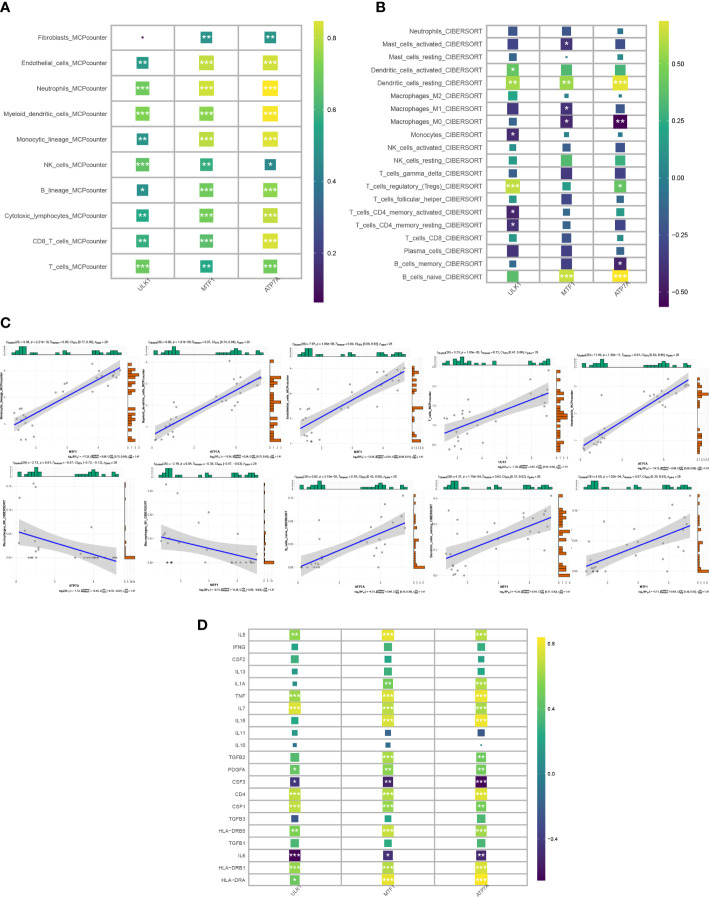
Correlation of candidate genes with immune cell infiltration. **(A, B)** Heat map of the correlation between 3 CRGs and immune cell infiltration in the MCPcounter and Cibersort algorithms. **(C)** Top 5 correlation plots in Cibersort and MCPcounter. **(D)** Heat map of correlation between candidate genes and inflammatory factors.

The “IOBR” package in R was used to conduct MCPcounter and CIBERSORT immune infiltration analysis on the 3 CRGs (**Figures 5A, B**). In MCPcounter, almost all immune cells showed a significant positive correlation with 3 CRGs, which were upregulated in the NS group. In CIBERSORT, dendritic cells resting showed a significant positive correlation with CRGs, T regulatory cells showed a positive correlation with ULK1 and ATP7A, and macrophages M0 showed a negative correlation with MTF1 and ATP7A. In addition, we generated correlation plots of the top 5 associations between CRGs and the corresponding immune cells using the CIBERSORT and MCPcounter algorithms (**Figure 5C**). In the analysis of inflammatory factors (**Figure 5D**), IL5, TNF, IL7, PDGFA, CD4, CSF1, HLA-DRB5, HLA-DRB1, HLA-DRA, and other factors showed significant positive correlations with 3 CRGs, while CSF3, IL6, and other factors showed significant negative correlations. Through the analysis of immune infiltration and inflammatory factors, it can be concluded that the CRGs have an extremely important influence on the immune and inflammatory reactions in the development of the disease.

### Consensus clustering analysis of HS

**Figure 6:**
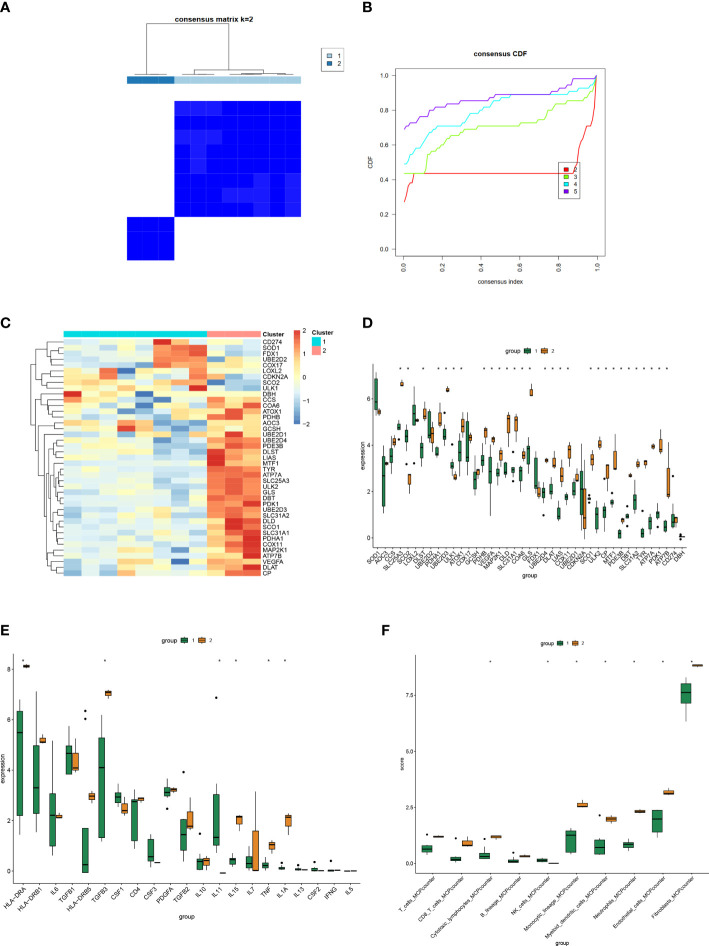
Consensus Clustering analysis of HS. **(A)** The circular manhattan (CM) plot exhibited the cluster at k = 2. **(B)** The area change under the empirical cumulative distribution function (CDF) curve when k=2-5. **(C, D)** Heat map and box plot of the expression distribution of Cuproptosis-related genes among different clusters. **(E, F)** Box plots of expression distribution of inflammatory factors and immune cell infiltration among different clusters. (*P < 0.05).

We performed consensus clustering analysis to group the dataset of HS, setting the maximum number of clusters to 5, and scoring the 2-5 clusters (**Figures 6A, B**). The results showed that when the number of clusters was k=2, the inter-cluster correlation was the lowest and the intra-cluster correlation was the highest, and the score was the highest. Therefore, we chose k=2 to group the dataset of HS and analyzed the gene expression of the two groups, and generated heatmaps and differential boxplots (**Figures 6C, D**). Finally, we further analyzed the expression differences of inflammatory factors and immune cells in the two groups (**Figures 6E, F**).

### Functional and pathway enrichment analysis

**Figure 7:**
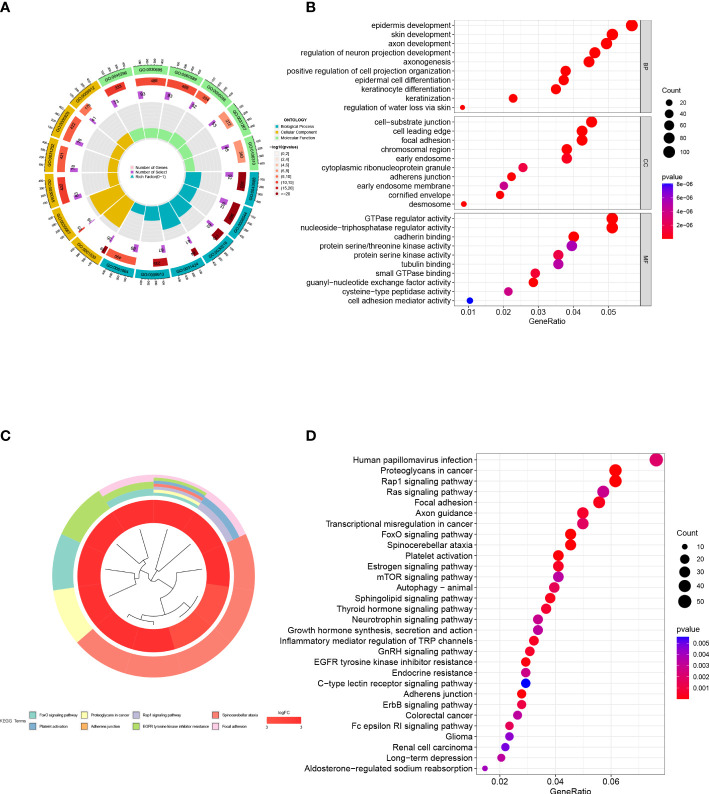
Functional enrichment analysis. **(A, B)** GO enrichment analysis of inter-subtype DEGs. **(C, D)** KEGG enrichment analysis of inter-subtype DEGs.

To investigate functional and pathway disparities between subtypes and unravel potential mechanisms of disease progression, we conducted GO and KEGG enrichment analysis using the differentially expressed genes identified between the two subtypes. In the BP category, the genes were mainly enriched in functions such as epidermis development, skin development, axon development, and regulation of neuron projection development. In the CC category, the genes were mainly enriched in functions such as cell-substrate junction, cell leading edge, focal adhesion, and chromosomal region. In the MF category, the genes were mainly enriched in functions such as GTPase regulator activity, nucleoside-triphosphatase regulator activity, and cadherin binding (**Figures 7A, B**). In the KEGG analysis, the genes were mainly enriched in metabolic pathways such as Human papillomavirus infection, Rap1 signaling pathway, Ras signaling pathway, and Focal adhesion (**Figures 7C, D**).

### Analysis of high cellular heterogeneity in human HS tissue through single-cell RNA-seq profiling

**Figure 8:**
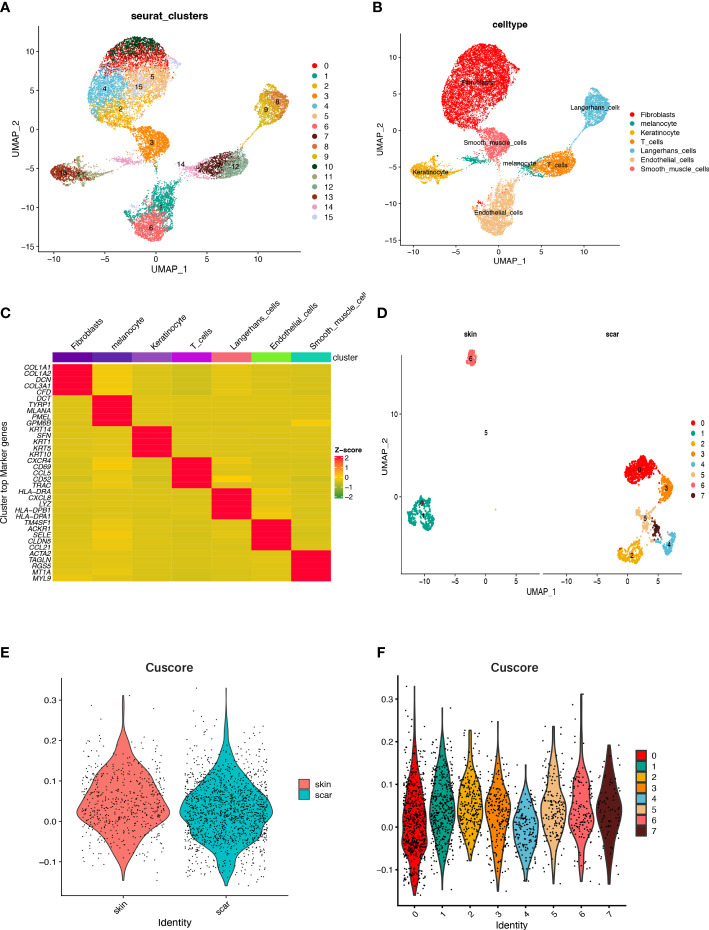
Cell populations and marker genes in HS and NS. **(A)** The cell clusters visualized by the dimensional reduction of t-SNE. **(B)** Cell subgroup annotation results. **(C)** A heatmap to display the TOP5 significant genes associated with each cell cluster, following differential analysis aimed at identifying marker genes. **(D)** Fibroblasts subpopulation clustering and dimensionality reduction. **(E)** The cuproptosis score of fibroblasts in HS and NS. **(F)** The cuproptosis score of the fibroblast subpopulations that were downscaled again.

We utilized scRNA-seq datasets containing 3 NS and 3 HS samples from the GEO database to unveil the intrinsic cellular heterogeneity within the skin tissue. After strict quality control, low-quality cells were excluded, and a total of 45095 cells were selected for subsequent analysis. Using dimensionality reduction clustering, we identified 16 cell clusters, which displayed high heterogeneity between different cell populations (**Figure 8A**). Based on marker genes identified in previous studies, we annotated 8 cell types, including Melanocytes (cluster 14, marked by MITF), Langerhans cells (clusters 8, 9, marked by Langerhans cells), T cells (clusters 7, 12, marked by PTPRC), Sweat gland cells (cluster 18, marked by SCGB1B2P, SCGB1D2), Fibroblasts (clusters 0, 2, 4, 5, 10, 15, marked by FBLN1, COL1A1, APOE, and APCDD1), Keratinocytes (clusters 11, 13, marked by KRT1 and KRT14), Smooth muscle cells (cluster 3, marked by ACTA2 and RGS5), and Endothelial cells (clusters 1, 6, marked by THBD and SELE) (**Figure 8B**). We then explored the TOP5 essential genes in seven previously annotated cell clusters and visualized the results (**Figure 8C**). **Figure 8D** also shows the proportion of fibroblasts in NS and HS. Further analysis of fibroblasts was performed by extracting them and reanalyzing them using tSNE and UMAP, which resulted in their re-clustering into 8 clusters. We scored the activity of cuproptosis in each cell using the AddModuleScore function, and found that the cuproptosis score of fibroblasts in NS was higher than that in HS (**Figure 8E**). Specifically, clusters 1 and 6 had the highest activity of cuproptosis (**Figure 8F**).

### Cell-cell communication

**Figure 9:**
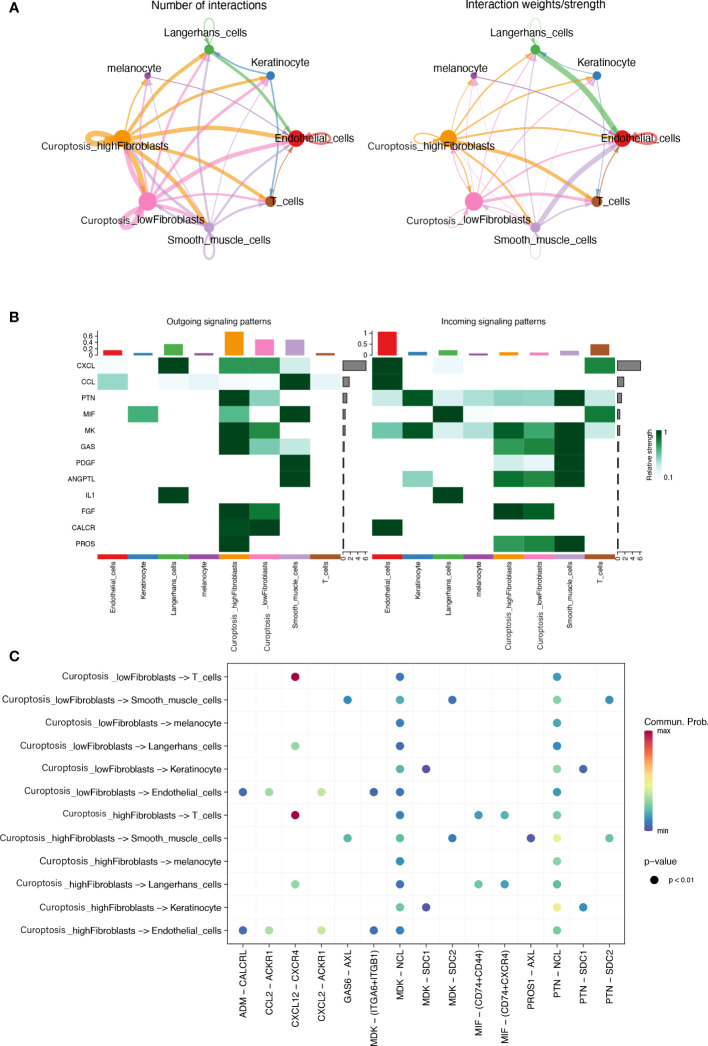
**(A)** An integrated cell-cell communication network mapped by the number and weight of interactions. **(B)** A visualization showing the relative strength of interactions, in terms of incoming and outgoing signals, across 8 different types of cells **(C)** A dot plot illustrating the signal pathways of incoming and outgoing interactions in two distinct subtypes of fibroblasts.

To decipher cell-cell signaling, we performed cell-cell communication analysis between different cell types using the “cellchat” R package. We classified fibroblasts into two types based on their previous cuproptosis activity scores, dividing them into high and low cuproptosis activity groups using the median value. Aggregated cell-cell communication networks were constructed based on interaction numbers (**Figure 9A**) and interaction weights (**Figure 9B**). The interaction strengths of cells transmitting and receiving signals were plotted in **Figure 9C**, which showed that fibroblasts played a crucial role as information senders in cell-cell communication. Low cuproptosis activity fibroblasts had weaker signal transmission strengths than high cuproptosis activity fibroblasts. We compared the interactions between the two types of fibroblasts and other cells. Compared to fibroblasts with low pathway activity, those with high pathway activity can engage in additional cell communication through the MIF pathway and with T cells and Langerhans cells. Additionally, they can communicate with smooth muscle cells via PROS1-AXL interactions.

### SCENIC transcription factor activity prediction and correlation analysis with CRGs

**Figure 10:**
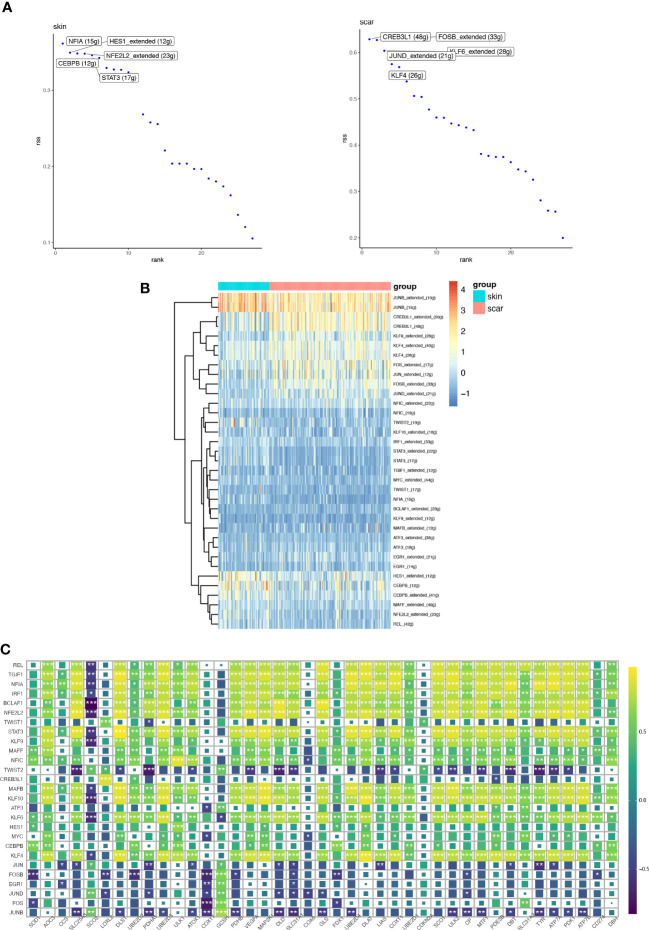
The transcription factors (TFs) and regulatory network involved in HS and NS. **(A)** The transcription factors regulating TOP5 activity in normal skin and hypertrophic scar fibroblasts. **(B)** Heatmaps showing the expression distribution of key transcription factors involved in differential transcription activity in HS and NS. **(C)** Correlation heatmap between key transcription factors involved in differential transcription activity and CRGs. (*P < 0.05, **P < 0.01, ***P < 0.001).

**Figures 10A, B** show the top 5 transcription factors with the highest activity, including NFIA, HES1, CEBPB, NFE2L2, and STAT3 in NS and CREB3L1, FOSB, JUND, KLF6, and KLF4 in HS. **Figure 10C** displays the differential expression of transcription factors between the two groups. We further analyzed the correlation between these transcription factors and CRGs at the bulk transcriptome level, and found that REL, TGIF1, NFIA, IRF1, BCLAF1, NFE2L2, STAT3, KLF9, MAFF, NFIC, MAFB, KLF10, KLF6, CEBPB, KLF4, and other transcription factors are positively correlated with most CRGs, suggesting a potential network of positive regulation. The remaining transcription factors are negatively correlated with most CRGs.

## Discussion

HS is a fibrous proliferative skin disease characterized by excessive deposition of extracellular matrix (ECM) and is also a chronic inflammatory disease. Its treatment, including laser therapy, surgery, and cryotherapy, is often challenging and there is no universally effective treatment (27). Early diagnosis and timely and effective targeted interventions are important, so there is a need to identify novel biomarkers that inhibit proliferation and reduce proliferative scar formation. Copper can cause aggregation of lipid acylated proteins and loss of iron-sulphur (Fe-S) cluster proteins by directly binding to the lipid acylated components of the tricarboxylic acid (TCA) cycle and increasing protein hydrotoxic stress, ultimately leading to cell death. This novel form of cell death is quite distinct from known forms of cell death (e.g. scorch death, apoptosis, ferroptosis and necroptosis) and is defined as Cuproptosis (10). The involvement of cuproptosis in the progression of fibrotic diseases (e.g. pulmonary fibrosis, myocardial fibrosis, oral mucosal fibrosis, etc.) has been demonstrated and is considered as a potential therapeutic strategy (11, 28, 29). However, the role of cuproptosis in skin fibrotic diseases has not been studied. Our study comprehensively analyzed the role of cuproptosis in the occurrence and development of HS for the first time through joint bulk and single-cell transcriptomics. We constructed a CRG diagnostic model for HS using machine learning and classified HS according to the cuproptosis pathway. We investigated the role of cuproptosis in cell differentiation, communication, and transcription factor regulation in fibroblasts.

In this study, 3 CRGs (ATP7A, ULK1, MTF1) were screened by differential gene analysis and machine learning algorithms (RF and SVM). ATP7A acts as a copper transporter protein and provides energy for copper transport. When intracellular copper levels are low, ATP7A recycles TGN and transports copper from the cytoplasm to the Golgi apparatus. Induction of ATP7A has been shown to cause renal fibrosis (30).MiRNA-1297 inhibits myocardial fibrosis by downregulating ULK1 (31),and short-chain fatty acids attenuate renal fibrosis via the HDAC2/ULK1 axis in metallotranscription factor-1 (MTF-1) has been shown to promote fibrosis (32). Saffronin ameliorates liver fibrosis by inhibiting HSC activation via the lnc-LFAR1/MTF-1/GDNF axis (33). These genes, which have not yet been found in HS, have significant potential for research. We validated the expression of the three genes in HS and NS by qRT-PCR and our results were consistent with expression differences in the RNA-seq dataset. We performed a multifactorial logistic regression approach to construct a diagnostic model using these three genes and validated the model as having high diagnostic value by bootstrap resampling.

Since immune cells and inflammatory factors play an important role in the development of HS (1), we applied the CIBERSORT,MCP algorithm to calculate the expression profile of immune cells in HS and to calculate the correlation of key genes associated with cuproptosis. Based on the results, we speculate that immune cells are extensively involved in the cuproptosis process in HS. CD4+ T cells and CD8+ T cells have been shown to potentially participate in hypertrophic scar formation. Tregs are beneficial to the heart by inhibiting excessive inflammatory responses and promoting early stable scar formation in cardiac injury (34). Macrophages undergo distinct phenotypic and functional changes at different stages of the pathogenesis of HS and are considered potential therapeutic targets (35). We hypothesize that cuproptosis may influence scar formation and development through the regulation of immune T cells and macrophages. MTF1, ULK1, ATP7A are associated with multiple inflammatory factors such as IL5, TNF, IL7, IL15, TGFB2, PDGFA, CSF3, CSF1, CD4, IL6, HLA-DRB1, HLA-DRA, etc., thus we found the excellent potential value of cuproptosis in the immunotherapy of HS. Since the clinical treatment of HS is often unsatisfactory, we divided HS into two clusters by cuproptosis pathway, in which immune cells such as cytotoxic lymphocytes, NK cells, myeloid dendritic cells, neutrophils were significantly different between the two subtypes (P value<0.05), demonstrating that they can be studied in depth as important targets for therapy.

As fibroblasts play an important role in HS (36), the changes in cuproptosis in skin fibroblasts were further explored in this study using the single cell dataset GSE156326. We divided fibroblasts into 8 cell clusters and calculated cuproptosis activity for each fibroblast. Based on our results, we found that cuproptosis activity was higher in fibroblasts from NS than HS, and we speculate that cuproptosis may alleviate skin fibrosis.

We compared the interaction of both types of fibroblasts with other cells, and high pathway activity fibroblasts could additionally communicate cellularly with Langerhans cells via the MIF pathway and T cells. MIF has been shown to have anti-fibrotic effects (37).The overexpression of protein S in lung cells reduces bleomycin-induced pulmonary fibrosis through the interaction between PROS1-AXL and communication with smooth muscle cells (38). Changes in cuproptosis activity in fibroblasts may affect other cell types and thus function through these specific receptor ligands. With these findings, we have further demonstrated that high cuproptosis activity can act as an inhibitor of fibrosis. We further analyzed the regulatory activity of transcription factors in skin fibroblasts and found that NFIA regulatory activity was highest in normal skin fibroblasts, and it has been shown that most DM rats developed retinopathy and lens fibrosis after inhibition of NFIA gene expression using RNA silencing (39). In HS fibroblasts, CREB3L1 regulatory activity was highest and CREB3L1 has been identified as a key transcription factor involved in HS myofibroblasts (21). The correlation between transcription factors with high activity in skin fibroblasts and CRGs was explored and from our results we know that CRGs are strongly positively and negatively correlated with a large number of transcription factors. Moreover, the transcription factors highly expressed in HS fibroblasts showed a negative correlation with CRGs, further confirming the potential of cuproptosis in alleviating skin fibrosis. This suggests that CRGs may act as potential target genes of transcription factors to influence phenotypic changes in skin fibroblasts.

While our study has yielded valuable insights into the relationship between certain CRGs and the development of HS, it is important to acknowledge certain limitations. One such limitation is the relatively small number of samples available in existing public databases, which may introduce statistical errors. Additionally, the clinical information available for these samples was limited, making it difficult to account for various analytical factors. It is clear that further research on CRGs is necessary to improve the overall predictive accuracy of our findings. Furthermore, there is a need for additional studies to investigate the molecular mechanisms underlying the influence of these CRGs on the formation and progression of HS.

## Conclusion

In conclusion, we explored the potential role of the cuproptosis in HS and provided a set of gene markers and constructed a diagnostic model, including ATP7A, ULK1, MTF1, which were validated by qRT-PCR in HS and NS and played a critical role in the development of HS. Subsequently, we further revealed the changes in the activity of cuproptosis in skin fibroblasts and its role in cell communication and transcription factor regulation activity through single-cell analysis. Therefore, our data provided insight into the development of more effective therapeutic interventions to improve the healing of HS.

## Data availability statement

The original contributions presented in the study are included in the article/supplementary material. Further inquiries can be directed to the corresponding authors.

## Ethics statement

The studies involving human participants were reviewed and approved by the Ethics Committee of the Fourth Military Medical University’s Xijing Hospital. The patients/participants provided their written informed consent to participate in this study.

## Author contributions

Conception and design: BYS, WL, and YZ; data curation and methodology: YZ, LC, and YP; analysis and interpretation of data: BG and ZC; writing of the manuscript: BYS and WL; review of the manuscript: LC, ZY, and BQS; study supervision: ZY and BQS. All authors contributed to the article and approved the submitted version.
